# Activity of pazopanib in *EWSR1-NFATC2* translocation-associated bone sarcoma

**DOI:** 10.18632/oncoscience.587

**Published:** 2023-09-20

**Authors:** Mohamed A. Gouda, Maria A. Zarzour, Ara A. Vaporciyan, Kalevi Kairemo, Hubert H. Chuang, Vivek Subbiah

**Affiliations:** ^1^Department of Investigational Cancer Therapeutics, The University of Texas MD Anderson Cancer Center, Houston, TX 77030, USA; ^2^Department of Sarcoma Medical Oncology, The University of Texas MD Anderson Cancer Center, Houston, TX 77030, USA; ^3^Department of Thoracic and Cardiovascular Surgery, The University of Texas MD Anderson Cancer Center, Houston, TX 77030, USA; ^4^Department of Nuclear Medicine, The University of Texas MD Anderson Cancer Center, Houston, TX 77030, USA; ^5^Sarah Cannon Research Institute, Nashville, TN 37203, USA

**Keywords:** pazopanib, precision oncology, sarcoma

## Abstract

Pazopanib is a multi-kinase inhibitor that is currently approved for treatment of advanced renal cell carcinoma and chemotherapy-refractory soft tissue sarcoma. In this case report, we discuss the case of a patient with a *EWSR1-NFATC2* fusion positive bone sarcoma who had exceptional tumor control through using pazopanib and surgery for an overall duration exceeding 5 years. We also review the literature on *EWSR1-NFATC2* translocation-associated sarcomas and use of pazopanib in bone sarcomas.

## INTRODUCTION

Bone sarcomas are a rare but aggressive group of cancers that strike adolescents and young adults in the prime of their lives [1–4]. There is a wide spectrum of histological diagnoses although osteosarcoma and Ewing sarcoma are the most common sub-types [5]. Beyond chemotherapeutic agents, unprecedented advances in immunotherapy and genomically targeted therapy that have conferred clinical benefit in many epithelial cancers have had minimal impact in the outlook of metastatic/relapsed bone sarcomas [6, 7]. Therefore, exploring other potential treatment options that can be used in bone sarcoma especially in the setting of molecularly driven therapeutics is needed.

Pazopanib is a multi-kinase inhibitor that works by targeting vascular endothelial growth factor receptor (VEGFR), platelet-derived growth factor receptor (PDGFR), fibroblast growth factor receptor (FGFR), and c-KIT; hence, inducing an antiangiogenic effect that leads to inhibition of tumor growth and apoptosis [8]. Pazopanib is currently approved by the United States Food and Drug Administration (FDA) for treatment of advanced renal cell carcinoma and advanced chemotherapy-refractory soft tissue sarcoma. However, it has been used off-label in many bone sarcomas and a few of them have been reported to derive clinical benefit. Molecular profiling and biomarkers may aid in understanding not only the diagnosis but also the underlying the response and/or resistance mechanisms [9].

In this case study, we report a patient with a *EWSR1*-*NFATC2* fusion positive bone sarcoma who had exceptional tumor control through using pazopanib and surgery for an overall duration exceeding 5 years. We also review the literature on *EWSR1*-*NFATC2* translocation-associated sarcomas and use of pazopanib in bone sarcomas.

## CASE PRESENTATION

**Figure 1:**
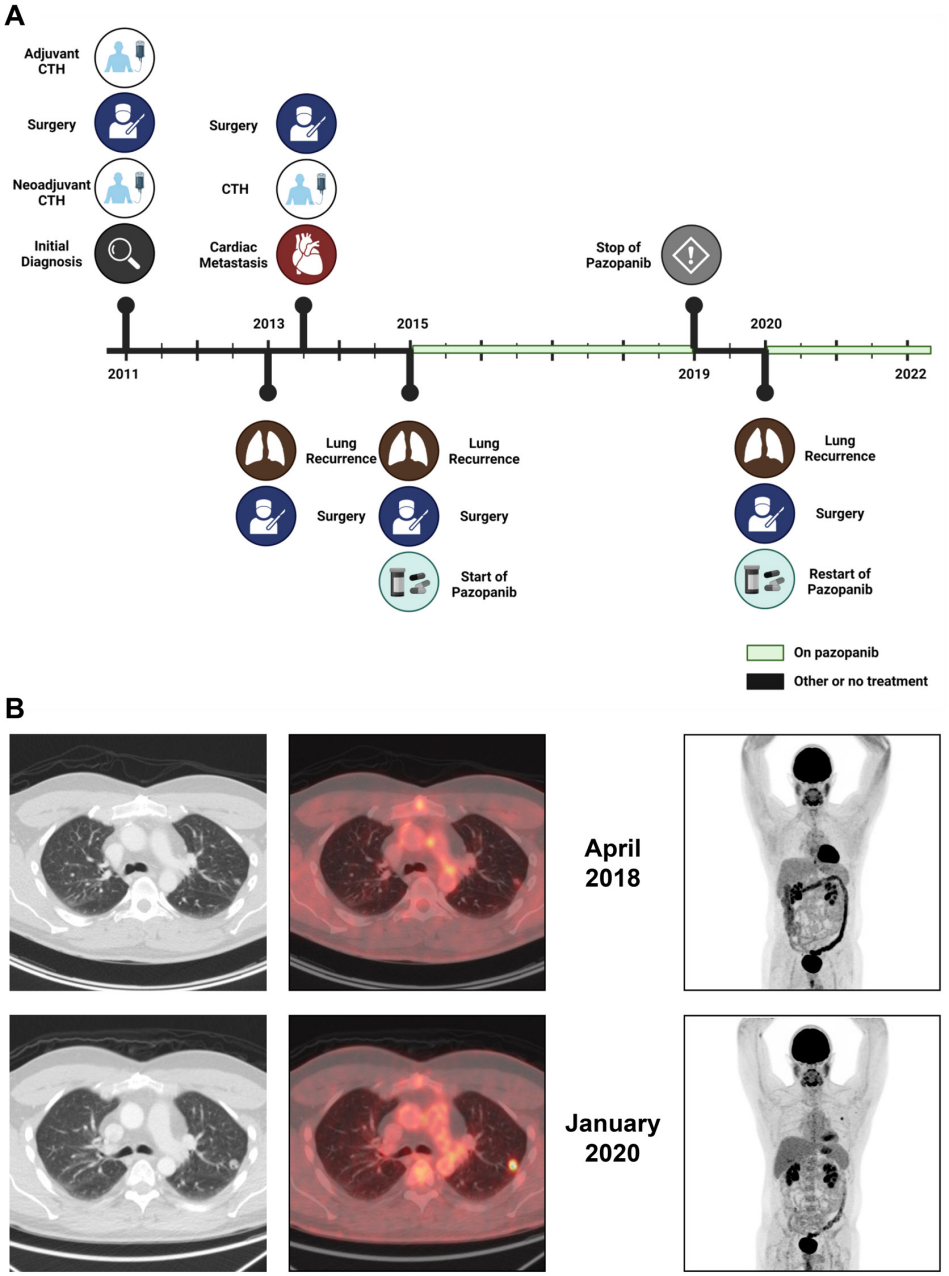
(**A**) Timeline showing patient’s treatment course. (**B**) Transaxial CT and PET slices at the lung metastasis level in the left lung on the left. Maximum intensity projection (MIP)-PET whole body images on the right. The upper row shows images in April 2018 when there was no evidence of metabolic active disease. The lower row images in January 2020 demonstrate a relapse in the left lung, which is seen transaxial images and as a tiny spot in the MIP-image.

A previously healthy male in his 30s initially presented with left leg mass. A biopsy was suggestive of high-grade bone sarcoma with small cell features. Patient received preoperative standard of care chemotherapy with vincristine, doxorubicin, cyclophosphamide, ifosfamide, and etoposide but showed minor tumor response and 10 to 20% necrosis in pathological analysis of the below knee amputation surgical specimen. He then received adjuvant chemotherapy with high dose ifosfamide alternating with doxorubicin and cisplatin but unfortunately developed lung recurrence after 2 years. Patient underwent wedge resection of lung metastasis that was consistent with the initial diagnosis of metastatic small round cell carcinoma. A comprehensive genomic profiling showed *EWSR1-NFATC2* fusion, *mTOR* E1799K mutation, and *TOP1* amplification. During postoperative imaging, cardiac metastasis was identified. Treatment with temozolomide and irinotecan was initiated but minimal response was observed, and patient was referred for surgical resection of the cardiac metastasis. The metastasis was attached only to a tricuspid valve chordae tendineae and an R0 resection was performed. Given poor response to previous therapies, patient was not offered any further adjuvant treatment and elected to active surveillance. Sixteen months later, a lung metastasis recurred, and he underwent another surgical resection. Based on multiple reports on response to pazopanib in patients with bone sarcomas and patient’s wish to pursue adjuvant treatment, therapy with pazopanib 800 mg orally daily was initiated one month after surgery. Patient’s disease remained under control for 5 years and after extensive discussion with patient weighing pros and cons of treatment discontinuation, pazopanib was stopped. A few months after cessation of pazopanib, patient developed disease progression to the lung which was again treated with upper lung wedge resection. Pathology showed metastatic round cell tumor. Patient had an uneventful post-operative course. After surgery, patient restarted pazopanib and to date continue to have a disease-free status for 30 months based on PET/CT regular follow up imaging (Figure 1).

## DISCUSSION

Pazopanib, a multi-kinase VEGF inhibitor, is currently FDA approved for advanced renal cell carcinoma and advanced soft tissue sarcoma; but limited evidence exists on its efficacy in bone sarcomas. Despite multiple preclinical studies supporting the biological rationale of using pazopanib [10–13], most clinical evidence comes from off label use reported in case reports and case series (Table 1). In addition, two phase 2 trials of pazopanib in bone sarcomas have shown promising results. Schulte et al. reported progression-free survival (PFS) at 12 weeks of 70% in an exploratory cohort of osteosarcomas receiving pazopanib and oral topotecan [median PFS = 4.5 months] [14]. Another phase 2 study (NCT01759303) suggested that 6 out of 12 patients with metastatic osteosarcoma had clinical benefit from pazopanib although the study was terminated early due to sponsor withdrawal [15].

**Table 1:** Summary of available evidence on the efficacy of pazopanib in osteosarcoma and Ewing sarcoma

Study	Design	Level of evidence^*^	Data summary	Biomarker
Aggerholm-Pedersen 2020 [16]	Case Series	Level 4	19 patients with bone tumors were treated with pazopanib (50% were osteosarcoma). Median OS was 11 months and PFS was 5.4 months. 13 patients (68%) had disease control (PR or SD)	N/A
Elete 2020 [17]	Case Series	Level 4	2 patients with relapsed osteosarcoma received pazopanib and achieved PR and SD	N/A
Tamura 2019 [18]	Case Report	Level 5	A patient with advanced Ewing sarcoma was treated with maintenance pazopanib after high-dose chemotherapy and radiotherapy and remained in near-complete remission after 1 year.	EWSR1 gene rearrangement by FISH
Seto 2019 [19]	Case Series	Level 4	A cohort of patients with bone sarcomas was included. Two patients with Ewing sarcoma (2/3) and other 2 with osteosarcoma (2/6) had SD for 6, 13, 6, and 9 months respectively	N/A
Takigami 2019 [20]	Case Report	Level 5	A patient with Ewing sarcoma//PNET had 5-months PFS following disease recurrence	EWSR1 gene rearrangement by FISH
Longhi 2018 [21]	Case Series	Level 4	15 patients with relapsed osteosarcoma received pazopanib. A clinical benefit (SD/PR) was observed in 9 patients (60%) with a median PFS of 6 months.	N/A
Mori 2018 [22]	Case Report	Level 5	A patient with metastatic extraosseous Ewing sarcoma showed marked response to pazopanib for a duration >26 months	EWS gene rearrangement by FISH
Stevens 2018 [23]	Case Report	Level 5	A patient with Ewing-like neoplasm of the parotid gland showed response to pazopanib after failed chemotherapy trial	EWSR1-KLF15 fusion by NGS
Umeda 2017 [9]	Case Series	Level 4	3 patients with recurrent osteosarcoma were treated with pazopanib. A patient was metastatic disease showed SD on pazopanib but decrease in ALP, a patient with localized disease was kept under control with adjuvant pazopanib for 6 months, and a patient with metastatic disease received pazopanib as adjuvant treatment with tumor control for 2 years.	N/A
Conry 2016 [24]	Case Report	Level 5	The study was done to evaluate zoledronic acid in osteosarcoma. One patient who developed metastasis showed PR after adding pazopanib and had long-term disease control.	N/A
Penel-Page 2015 [25]	Case Report	Level 5	A retrospective analysis of off-label use of targeted therapies in osteosarcoma showed 1 patient who was treated with pazopanib but showed PD	
Attia 2015 [26]	Case Report	Level 5	A patient with heavily pretreated extraosseous Ewing sarcoma shoed disease control on pazopanib	EWSR1 gene rearrangement by FSH ERCC1 and RRM1 high by RT-PCR PTEN positive by IHC
Alcindor 2014 [27]	Case Report	Level 5	A patient with recurrent Ewing sarcoma showed PD on pazopanib	N/A
Safwat 2014 [28]	Case Series	Level 4	3 patients with metastatic osteosarcoma showed 2 SD and 1 PR along with clinical improvement	N/A
Yamamoto 2014 [29]	Case Report	Level 5	A patient with extraosseous Ewing sarcoma was treated with pazopanib but showed PD	EWSR1-FL1 fusion by FISH

Abbreviations: OR: Overall survival; PFS: Progression-free survival; PR: Partial response; SD: Stable disease; PD: Progressive disease. ^*^Reported levels of evidence are per American Society of Plastic Surgeons (ASPS) guidelines accessible at https://www.plasticsurgery.org/documents/medical-professionals/health-policy/evidence-practice/ASPS-Rating-Scale-March-2011.pdf.

Our patient showed a dramatic long-term response to pazopanib following multiple failed trials of chemotherapy that were followed with disease recurrence. Interruption of pazopanib led to interval disease progression which validates the contribution of pazopanib to long-term disease control. A review of literature shows the definitive benefit of pazopanib in anecdotal cases of bone sarcomas (Table 1). However, biomarker-based reporting has been only presented in few studies. Molecular profiling has shown a great potential in guiding treatment decisions including those in patients with bone tumors [30, 31]. A study by Egas-Bejar et al. [7] suggested that mutations in PI3K/PTEN/mTOR pathway are not uncommon in patients with osteosarcoma. Not only can genetic testing identify actionable alterations, but it can also help to molecularly characterize tumors’ behavior and establish prognostic subgrouping usable in clinical management [7]. In our case, the patient’s tumor harbored *EWSR1-NFATC2 fusion*, *mTOR* E1799K mutation, and *TOP1* amplification. *EWSR1* encodes for the EWS protein which plays a pivotal role in gene transcription. Alterations in *EWSR1* gene, including gene rearrangements, have been commonly linked to cases with bone sarcomas via aberrant dysregulation of gene transcription leading to uncontrolled cellular growth and survival [32]. Most fusion partners that have been described belong to the ETS family of genes, including *FLI1* and *ERG* genes, but more recently interest has grown in *EWSR1*-non-ETS fusions including *EWSR1-NFATC2*. In fact, newer evidence suggests that sarcomas with that *EWSR1-NFATC2* have distinct tumor characteristics and should be considered as a separate disease entity from other bone sarcomas (Table 2). Translocation-associated small round cell sarcoma with *EWSR1-NFATC2* fusion has been described to be resistant to conventional Ewing sarcoma chemotherapy [33]. A multiscale-omic analysis revealed upregulation of the mTOR pathway in those patients which presents another chance for therapeutic targetability in the era of precision oncology [34]. Interestingly, *mTOR* E1799K mutation was also observed in our patient. mTOR is an atypical protein kinase that is proposed to be linked to the PI3K signaling pathway dysregulation of which has been postulated as a potential mechanism for oncogenesis [35, 36]. In addition to *EWSR1* and *mTOR*, *TOP1* amplifications were also identified in our patient and previously hypothesized to associate with more aggressive tumors in patients with melanoma and responses to TOP1 inhibitors including topotecan and irinotecan [37].

**Table 2:** Summary of reported *EWSR1-NFATC2* malignancies in literature and response to therapy whenever applicable

Reference	Summary
Tsuchie 2022 [38]	*EWSR1-NFATC2* was detected in a 39-year-old woman with soft tissue round cell sarcoma.
Brcic 2022 [39]	*EWSR1-NFATC2* was detected in three patients with different neoplasms (1 complex cystic bone lesion and 2 sarcomas).
Seligson 2021 [34]	Fourteen cases with *EWSR1-NFATC2* fusions were identified and shown to possess an upregulation of mTOR pathway. A 58-year-old male patient with metastatic *EWSR1-NFATC2* fusion positive sarcoma had 47 months of stable disease with combined mTOR and VEGF inhibition.
Dashti 2021 [40]	*EWSR1-NFATC2* fusion was detected in three cases with sarcoma.
Yoshida 2020 [41]	*NKX3-1* was expressed in 9 out of 11 cases with *EWSR1-NFATC2* sarcomas
Perret 2020 [42]	*EWSR1-NFATC2* fusion was detected in four sarcoma patients (one inoperable who received chemotherapy and radiotherapy and three who received neoadjuvant chemotherapy with necrosis <90% in surgical specimen). Tumors were positive for AGGRECAN which could be used for diagnostic purposes.
Tsuda 2020 [43]	*EWSR1/FUS-NFATC2* fusions positive sarcoma were detected in 10 out of 226 patients with Ewing sarcoma
Yau 2019 [44]	*EWSR1-NFATC2* fusion was detected in a 43-year-old man with bone sarcoma with minimal response to neoadjuvant Ewing sarcoma protocol.
Koelsche 2019 [45]	In five cases with undifferentiated round cell sarcomas and *EWSR1-NFATC2* fusion, there was a distinct methylation pattern different from that of Ewing sarcoma and other sarcoma subtypes
Wang 2019 [33]	Six sarcoma patients with *EWSR1-NFATC2* fusion had poor response to preoperative chemotherapy (only one patient who had concurrent IMRT had 90% necrosis and slight reduction in tumor size)
Bode-Lesniewska 2019 [46]	*EWSR1-NFATC2* fusion was detected in three patients with mesenchymal tumors (initially diagnosed as sclerosing epithelioid fibrosarcoma, myoepithelial tumor, and extraskeletal myxoid chondrosarcoma
Diaz-Perez 2019 [47]	*EWSR1-NFATC2* fusion was detected in three cases with round cell sarcoma and had poor response to Ewing sarcoma chemotherapy
Watson 2018 [48]	Round cell sarcomas can be molecularly subgrouped using transcriptomics including the distinct characteristics of *EWSR1-NFATC2* fusion
Machado 2018 [49]	*EWSR1-NFATC2* was detected in a case with undifferentiated small round cell tumor
Toki 2018 [50]	In a case with *EWSR1-NFATC2* sarcoma, there was a co-expression of *PAX7* and *NKX2-2*
Cohen 2018 [51]	*EWSR1-NFATC2* fusion was detected in a 24-year-old woman with soft tissue tumor that had an epithelioid round cell morphology (initially diagnosed as extraskeletal Ewing Sarcoma). No response to preoperative chemotherapy was observed in surgical posttreatment specimen.
Charville 2017 [52]	*EWSR1-NFATC2* fusion can mediate *PAX7* expression in Ewing sarcoma
Baldauf 2017 [53]	*EWSR1-NFATC2* translocated sarcomas are distinct from *EWSR1-ETS* translocated Ewing sarcoma
Kinkor 2014 [54]	*EWSR1-NFATC2* fusion was detected in two cases with Ewing-like sarcoma which were aggressive and chemoresistant
Sadri 2014 [55]	*EWSR1-NFATC2* fusion was detected in a 30-year-old male patient with malignant round cell tumor of the bone who did not tolerate adjuvant chemotherapy
Eang 2012 [56]	Two Ewing sarcoma cases with *EWSR1-NFATC2* fusion had weak reactivity to anti-FLI1 antibody and weak or no reactivity to anti-ERG antibody
Szuhai 2009 [57]	*EWSR1-NFATC2* fusion was identified and cloned in a variant of Ewing sarcoma
Romeo 2012 [58]	One case with myoepithelioma like pattern harbored *EWSR1-NFATC2* fusion
Arbajian 2013 [59]	*EWSR1-NFATC2* fusion was detected in a case of hemangioma of the bone
Mantilla 2019 [60]	*EWSR1-NFATC2* fusion was detected in a patient with ossifying fibromyxoid tumor
Hung 2021 [61]	*EWSR1-NFATC2* was detected in 3 cases out of 9 with simple bone cysts which were negative for *NKX3.1* and *NKX3.2* Three
Ong 2021 [62]	*EWSR1-NFATC2* fusion was detected in 6 out of 9 simple bone cysts and 3 out of 12 benign vascular tumors.
Pizem 2021 [63]	*EWSR1-NFATC2* fusion was detected in two cases with simple bone cysts.
Brcic 2022 [39]	*EWSR1-NFATC2* was detected in three patients with different neoplasms (1 complex cystic bone lesion and 2 sarcomas).
Makise 2021 [64]	*EWSR1-NFATC2* fusion was detected in a 26-year-old woman with Ewing-like adamantinoma.

With its multi-kinase activity, including actions on VEGF, PDGFR, FGFR, and KIT, pazopanib leads to a desirable inhibition of tumor growth which antagonizes the impact of such tumor-promoting alterations and possibly explain the derived clinical benefit in our patient. This benefit is probably derived from the action on VEGF which has been reported to be upregulated in patients with sarcoma [65, 66]. Since *mTOR* overactivation might have been the case in our patient either through the upregulation *by EWSR1-NFATC2* fusion [34] or the co-occurring *mTOR 1799K* mutation, it is possible that such activation might have led to increased VEGF given the connection between both pathways [67]; which in turn yielded the tumor responsive to pazopanib. Moreover, there is at least some evidence that other EWS fusions, e.g., EWS-FLI, are directly associated with increase in VEGF and tumor-associated angiogenesis [65, 66]. Responses to pazopanib inhibition have been reported in non-bone sarcomas with *EWS-ATF1* and *EWSR1-CREB1* fusions [68, 69]. Interestingly, a cell line Hewga-CCS was established from a clear cell sarcoma harbored the type 2 *EWS-ATF1* transcript. In the pre-clinical studies reported, pazopanib suppressed the growth of these cell lines both *in vivo* and *in vitro*. Intriguingly, A phospho-receptor tyrosine kinase array revealed phosphorylation of c-MET, but not of VEGFR in these models and ensuing experiments revealed that pazopanib exerted antitumor effects through the inhibition of HGF/c-MET signaling [69]. It is possible that other related fusions, including the *EWSR1-NFATC2* fusion detected in our patient, may confer a shared inherent potential sensitivity to pazopanib via its association with VEGF and perhaps other pathways like HGF-c-MET.

In brief, this case, in accordance with previously reported evidence, provides proof of activity of pazopanib in *EWSR1-NFATC2* positive sarcoma. The report shows that pazopanib when administered in an adjuvant capacity demonstrated its effectiveness in preventing or delaying the progression of additional metastasis. Nevertheless, due to the adjuvant nature of the treatment, it remains uncertain whether this approach would have resulted in tumor shrinkage. Further pre-clinical studies and clinical studies using pazopanib in *EWSR1-NFATC2* sarcomas are warranted.
